# Transforming Mental Health for Transgender and Gender-Diverse Young Adults Using Interventions to Drive Equity (TransHealthGUIDE): Protocol for a Digital Randomized Controlled Trial

**DOI:** 10.2196/78619

**Published:** 2025-11-11

**Authors:** Sari L Reisner, S Wilson Cole, Alex S Keuroghlian, Sabra L Katz-Wise, Cassandra Morrow, Kaiden Kane, Caitlynn Feng, Rena Xu

**Affiliations:** 1 Department of Epidemiology University of Michigan School of Public Health Ann Arbor, MI United States; 2 Health, Behavior and Society Johns Hopkins Bloomberg School of Public Health Baltimore, MD United States; 3 Queery Research Consulting Glen Burnie, MD United States; 4 Department of Psychiatry Massachusetts General Hospital Boston, MA United States; 5 Department of Psychiatry Harvard Medical School Boston, MA United States; 6 Division of Adolescent/Young Adult Medicine Boston Children's Hospital Boston, MA United States; 7 Department of Pediatrics Harvard Medical School Boston, MA United States; 8 Department of Social and Behavioral Sciences Harvard T.H. Chan School of Public Health Boston, MA United States; 9 Department of Urology Boston Children's Hospital Boston, MA United States; 10 Department of Surgery Harvard Medical School Boston, MA United States

**Keywords:** transgender, mental health, young adult, randomized controlled trial, mobile apps

## Abstract

**Background:**

Transgender, nonbinary, and gender diverse (TGD) young adults face a disproportionately high mental health burden, including 2- to 4-fold increased risk of suicidality and depressive distress compared to their cisgender peers. Family and community support are protective factors that may mitigate these adverse outcomes, representing key targets for mental health interventions.

**Objective:**

This randomized controlled trial (RCT) evaluates the effectiveness of TransHealthGUIDE, a novel digital, app-based education and support intervention aimed at reducing past 3-month suicidal ideation and past 2-week depressive distress among TGD young adults. Participants are randomized 1:1 to an immediate intervention arm or a 6-month deferred intervention arm (waitlist control).

**Methods:**

From 2021 to 2024, a multidisciplinary team of academic and community partners conducted formative research and codeveloped a digital app containing 33 self-paced modules, communication, and interactive resources. The content is grounded in the Social Ecological Suicide Prevention Model and the Gender Minority Stress and Resilience Framework, designed to enhance family support and community connection for TGD young adults and their families. TGD young adults (ages 18-24 years) and their caregivers are prospectively enrolled and randomized in 10 US states (California, Connecticut, Illinois, Maryland, Massachusetts, New Jersey, New York, Oregon, Vermont, and Washington, DC). Participants complete self-reported surveys at baseline, 6 months, and 12 months, assessing sociodemographics, family support, community connection, suicidal ideation, depressive distress, and intervention acceptability. App usage and engagement are tracked via metadata. The target sample size is 500 TGD young adults, with 50% (n=250) identifying as people of color and 125 TGD young adult-caregiver dyads.

**Results:**

TGD young adult and caregiver communities, including our community advisory board (CAB), have guided all aspects of the project: study design (eg, waitlist control), survey development (eg, self-reported rather than clinician-administered suicidality measures and inclusion of social justice activism as a form of support), intervention content (eg, text, infographics, interviews with TGD people and caregivers, and simulations), and digital app safety features (eg, a quick-exit button for privacy). Recruitment and study screening began on January 21, 2025. As of May 2, 2025, a total of 149 individuals completed a study screener, with 132 (88.6%) of 149 found eligible. Enrollment visits began on April 23, 2025. As of May 2, 2025, five TGD young adults have been enrolled. Ongoing engagement with the CAB will inform iterative improvements throughout the trial.

**Conclusions:**

Scalable, app-based interventions tailored to meet the needs of TGD young people hold promise for population-level mental health impact. This RCT—targeting suicidality and depressive distress via education and support for both TGD young adults and their caregivers—addresses a critical gap in mental health interventions for this underserved population.

**Trial Registration:**

ClinicalTrials.gov NCT06177600; https://clinicaltrials.gov/study/NCT06177600

**International Registered Report Identifier (IRRID):**

DERR1-10.2196/78619

## Introduction

### Background

Transgender, nonbinary, and gender diverse (TGD) individuals experience multiple forms of interpersonal and structural stigma and face intersectional gender-, race-, and socioeconomic-based minority stress at various levels [1-4], including within their own families [5-8], resulting in life-threatening health disparities. Suicide, one of the top 10 causes of death in the United States and the second leading cause of death among young adults [9,10], is 2-4 times more likely to affect TGD young adults compared to their cisgender peers [11,12]. Rates of depressive distress are similarly elevated among TGD young adults [11-13], as are other mental health conditions such as nonsuicidal self-injury, anxiety, and substance use [11-14].

Compounding these disparities, TGD individuals are more likely than the general population to identify as Black or Hispanic, and they frequently experience race-based health inequities [15]. For Black and Hispanic communities, which already face a disproportionately high burden of suicide compared to the general population [16], these intersecting vulnerabilities further increase risk for Black and Hispanic TGD individuals.

There is an urgent need for evidence-based interventions that address the unique mental health challenges faced by TGD young adults. Research shows that family functioning and support—including communication, satisfaction, and acceptance—are protective factors for TGD individuals’ mental health [5,6,17,18]. Additionally, a strong sense of community connectedness is associated with improved mental health outcomes [19-21]. These findings emphasize the crucial role of supportive relational contexts—within families and communities—in promoting TGD individuals’ well-being. Self-guided digital mental health interventions, including web- and smartphone-based apps, have demonstrated effectiveness in reducing depression and suicidality among young people [22,23]. They have also been shown to increase perceived social support [24] and are generally well-accepted by individuals experiencing mental health concerns [25]. However, to our knowledge, no evidence-based digital app intervention currently exists that is designed to reduce depression and suicidality among TGD young adults, while also engaging their caregivers or families in the process.

### Study Objective and Aims

This prospective randomized controlled trial (RCT) evaluates the effectiveness of an educational and supportive app-based digital intervention in reducing suicidal ideation and depressive distress among TGD young adults across 10 US states. TransHealthGUIDE (Transforming Mental Health for Transgender and Gender-Diverse Young Adults Using Interventions to Drive Equity) is a co-designed digital app developed in partnership with TGD communities. The intervention engages, empowers, and fosters connections between TGD young adults and their families.

The primary aim of this study is to test the effectiveness of a digital app-based intervention in reducing past 3-month suicidal ideation and past 2-week depressive distress among TGD young adults and their caregivers, by comparing participants randomized 1:1 to an immediate intervention arm versus a 6-month deferred intervention arm (waitlist control).

The secondary aims are (1) to assess the impact of the intervention on family functioning (past 3-month family communication, satisfaction, and acceptance) and on community belonging and connection for TGD young adults and their caregivers, and (2) to examine the dyadic relationship dynamics between TGD young adults and their caregivers participating in the intervention.

## Methods

### Study Design

**Figure 1:**
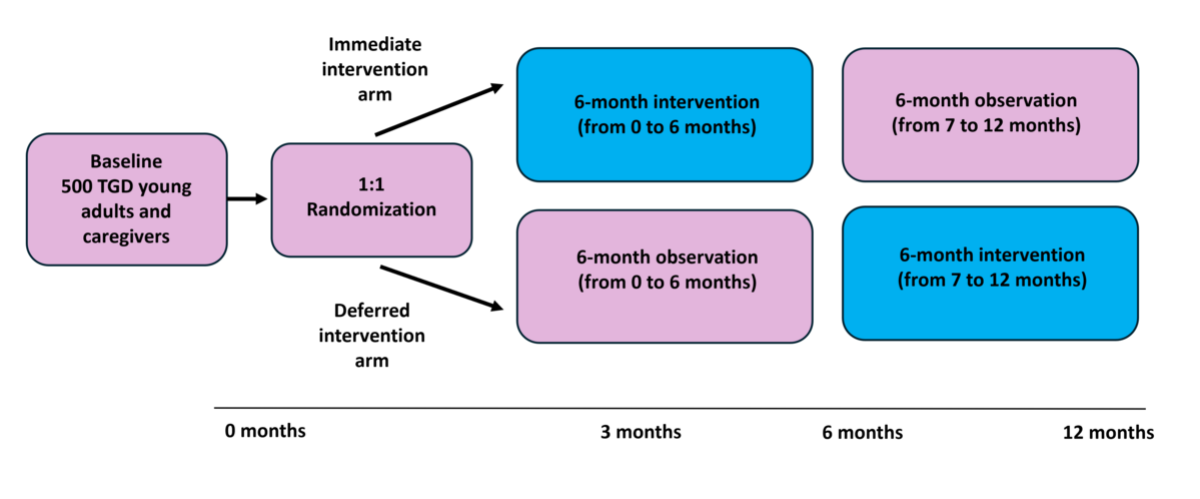
TransHealthGUIDE (Transforming Mental Health for Transgender and Gender-Diverse Young Adults Using Interventions to Drive Equity) study schema. Target sample size is 500 TGD young adults, with one-quarter (125 participants) in a dyad with a caregiver (target sample size of 125 caregivers). TGD: transgender, nonbinary, and gender diverse.

This is a prospective, digital RCT in which TGD young adults and their caregivers are randomized 1:1 to either an immediate intervention arm (a 6-month active intervention period followed by 6 months of observation) or a deferred intervention arm (a 6-month waitlist period followed by active intervention). Each participant is enrolled in the trial for a total of 12 months (see Figure 1). The total study duration is approximately 2 years.

Participants in the immediate intervention arm receive full access to the TransHealthGUIDE digital app for the first 6 months. This is followed by a 6-month observation period during which they retain access to the app but receive no new intervention content or engagement prompts. Participants in the deferred intervention arm receive limited access to the app for the first 6 months, consisting primarily of curated local, regional, and national support resources. At the 6-month mark, participants in the deferred arm are granted full access to the digital app for the remainder of their participation.

Survey assessments are administered at baseline, 6 months, and 12 months to evaluate primary outcomes (past 3-month suicidal ideation and past 2-week depressive distress) as well as secondary measures.

### Project History and Multidisciplinary Team

In 2021, the National Institutes of Health (NIH) Common Fund launched the “Transformative Research to Address Health Disparities and Advance Health Equity” initiative. The goal of this initiative was to support innovative, translational research projects aimed at preventing, reducing, or eliminating health disparities and advancing health equity [26]. This grant mechanism specifically supported groundbreaking interventions targeting one or more NIH-designated populations that experience health disparities in the United States. Notably, pilot data were not required; instead, emphasis was placed on the development and implementation of novel, creative interventions.

Our multidisciplinary team chose to focus on the persistent mental health disparities affecting TGD young adults, particularly elevated rates of suicidal ideation and depressive distress. To address this urgent need, we conducted extensive formative research to develop a digital, app-based intervention designed to reduce these disparities by increasing family support and community connection.

The intervention was developed through a community-engaged process and reflects the combined expertise of a multidisciplinary team with backgrounds in TGD health, educational and curriculum design, sociobehavioral interventions, digital health development, clinical trials, psychiatry and mental health, young adult developmental psychology, family systems, and TGD lived experience. TGD young adults and caregivers were actively involved throughout the development process, ensuring that the intervention was relevant, affirming, and grounded in real-world experiences.

### Conceptual Framework

Digital interventions grounded in evidence-informed conceptual frameworks are more likely to be effective than those lacking theoretical design [27]. TransHealthGUIDE is informed by several evidence-based models of suicide prevention and mental health promotion tailored to meet the needs of TGD young adults.

First, the intervention aligns with the Social Ecological Suicide Prevention Model [28], addressing the multiple levels of influence—individual, interpersonal, and structural—that shape TGD individuals’ experiences. Intervention content focuses on helping users navigate and cope with challenges across various contexts, including families, schools, and health care settings, while targeting key drivers of suicidality such as bullying, misgendering, and systemic exclusion.

Second, TransHealthGUIDE draws from the Gender Minority Stress and Resilience Framework [29,30], which emphasizes the impact of societal stigma, discrimination, and oppression on TGD individuals’ mental health [31]. The intervention also emphasizes resilience, using a strengths-based approach to bolster self-efficacy and support-seeking behaviors [32].

Finally, the intervention incorporates an intersectional lens by explicitly addressing intersectionality [33] and centering the experiences of individuals with multiply marginalized and intersecting identities, including those related to race, ethnicity, disability, body size, gender identity, sexual orientation, neurodivergence, and religion. This intersectional framework ensures that the content remains relevant and affirming across a broad spectrum of identities and lived experiences.

### Community Consultations and CAB

In 2022, our team conducted extensive community consultations, engaging with more than 20 key stakeholders to better understand the mental health and support needs of TGD young adults and their families, identify potential intervention strategies, and establish partnerships to support future recruitment and implementation efforts. These consultations included national organizations such as the World Professional Association for Transgender Health (WPATH) and TransLine, as well as local organizations serving TGD young adults and their families within communities across the United States.

In 2023, we formally established a community advisory board (CAB) consisting of 16 members: 7 TGD young adult community members and 9 caregivers and health care providers, including mental health professionals. The CAB is geographically diverse, representing the Northeast, Mid-Atlantic, Midwest, South, and West regions, and 7 (43.8%) of 16 are people of color.

Our CAB has played a central role in guiding the project. Their input has shaped intervention content, informed recruitment and retention strategies, and contributed to the critical decisions regarding implementation of the RCT. Their ongoing engagement ensures the intervention remains grounded in lived experience and community priorities.

### Formative Research

We conducted extensive mixed methods formative research to inform the study design and procedures, intervention content, digital app features and aesthetics, recruitment and retention strategies, and dissemination plans to ensure high acceptability and relevance to our target population.

Between April and August 2022, we conducted 33 qualitative interviews with a purposive sample of 13 medical and mental health care providers, 14 caregivers, and 6 TGD young adults, recruited through community partners and the team’s professional and personal networks. These interviews highlighted a significant unmet need for support and education among both TGD young adults and caregivers, including a desire for accessible, accurate, expert-informed information [34].

In August 2022, we also conducted a one-time survey with 104 young adults, of whom 48 (46.2%) were people of color, recruited via the Prolific.com platform. Eligible participants were US residents aged 18-25 years who identified as TGD. The survey included both closed- and open-ended questions to explore how TGD young adults seek gender- and health-related information online. Findings revealed a high prevalence of depressive distress, with 44 (42.3%) of 104 individuals meeting criteria for clinically elevated symptoms. Low family support was a strong correlate of depressive distress, underscoring the need for interventions aimed at strengthening relationships between TGD young adults and their caregivers [18]. Participants also expressed a strong preference for receiving information through personal stories and lived experiences of other TGD people and families—an insight that directly shaped the narrative and multimedia components of our intervention [35].

Overall, our formative research revealed substantial educational and emotional support needs among TGD young adults and families, particularly regarding navigation of familial and relational dynamics and the search for affirming community connections.

### Intervention Development and Content

The TransHealthGUIDE digital interactive app was developed between 2023 and 2024, drawing on insights from formative research, community consultations, ongoing CAB feedback, and the multidisciplinary team’s expertise in TGD research and clinical care. The team brought together diverse areas of expertise, including provider education, digital health innovation, and computational informatics in mental health (RX); TGD research methods, clinical trials, and community-based sociobehavioral interventions (SLK-W, SLR, and SWC); suicide prevention, psychiatric care, and mental health intervention development (ASK); TGD young adult development, family systems theory, and developmental psychology (SLK-W); and instructional and curriculum design tailored to TGD populations (SWC). App design and development were carried out in partnership with Vessel Partners, a company that specializes in creating and testing app-based interventions for sociobehavioral research, with experience supporting research-grade digital tools.

The app’s educational content was developed specifically for this intervention and includes text-based modules offering psychoeducational content, video narratives featuring real-life stories from TGD young adults and caregivers, and simulation-based (scripted and acted) videos, which used professional actors to portray challenging conversations between TGD young adults and caregivers, modeling best practices for communication and support.

The app includes 33 self-paced educational modules focused on reducing depressive symptoms and suicidal ideation among TGD young adults, with some modules specifically designed for caregivers. Module summaries, including titles, intended audience, and learning objectives, are provided in Table 1. Participants can access and complete modules on smartphones, tablets, or computers, in any order, and may skip or repeat modules. Each module lists an estimated completion time—most under 15 minutes—to support flexible, manageable engagement.

**Table 1:** Overview of the TransHealthGUIDE^a^ intervention modules, target audience, and learning objectives.

Module number	Module titles	Audience	Learning objectives (by the end of the lesson module...)
1	Introduction	TGD^b^ young adults and caregivers	TGD young adults and caregivers will be welcomed and will recognize the various functionalities and features of the app.
2	Introduction: Caregivers	Caregivers	Caregivers will be able to name and recognize similarities and differences between their experiences and the caregivers highlighted in the video. Caregivers will gain insights from the video about tools they can use in their own parenting or caregiving journey.
3	Terms & Definitions	TGD young adults and caregivers	TGD young adults and caregivers will be able to define foundational sexual orientation, gender identity, and expression (SOGIE) terminology and concepts.
4	Gender Identity	TGD young adults and caregivers	TGD young adults and caregivers will be able to recognize the complexities of gender both in the present and historically; identify and name gender identities under the transgender, nonbinary, and gender expansive identity umbrellas; and foster open and empathetic communication between young people and their caregivers about diverse gender identities.
5	Names	TGD young adults and caregivers	TGD young adults will gain skills in choosing a name (if they have not already) and how to discuss name changes with their caregiver. TGD young adults and caregivers will gain the skills to locate and engage with systems and legal processes to affirm and legalize names for TGD young adults.
6	Pronouns	TGD young adults	TGD young adults will gain the skills to effectively communicate their pronouns and leverage support in settings such as home or school.
7	Gender Expression	TGD young adults and caregivers	TGD young adults and caregivers will recognize the complexities of gender expression and gain skills to respectfully engage with people outside of gender assumptions based on appearance. TGD young adults will name and recognize similarities and differences between their experiences of and desires for gender expression and those in the videos.
8	Imagining My Future	TGD young adults	TGD young adults will build a foundation of skills to foster self-compassion and care when facing challenging situations. TGD young adults will develop goal-setting skills and write gender- and relationship-related goals for the next 3 months.
9	Goals: Self & Youth	Caregivers	Caregivers will build a foundation of skills to foster self-compassion and generate a circle of support for themselves and their young person. They will define and write goals for their own growth and around engaging positively and affirmatively with their young person.
10	Dysphoria	TGD young adults	TGD young adults will gain skills to recognize and address dysphoria, as well as access support.
11	What to Expect/Transitions	TGD young adults	TGD young adults will be able to relate to the experiences shared by TGD individuals in various stages, identify key elements of psychological and social transition, and gain insights into the personal psychological benefits of the transition process.
12	Intersectionality: Identities, Communities, and Me	TGD young adults and caregivers	TGD young adults and caregivers will be able to describe intersectionality and how different identities have different levels of marginalization in society. TGD young adults and caregivers will be able to describe how stigma and discrimination impact TGD people, especially those with multiple, marginalized identities.
13	Communicating with Caregivers	TGD young adults	TGD young people will gain skills to communicate effectively, establish boundaries, and improve their relationships with their caregivers.
14	Communicating with Your TGD Loved One	Caregivers	Caregivers will gain skills to facilitate improved communication with their young person, including how to handle mistakes, begin again, and build healthy relationships with their young person.
15	Struggling to Accept	Caregivers	Caregivers will gain skills to acknowledge and appropriately process their own emotions, such as grief, confusion, and anger, without burdening their young person. They will also gain insights on how to provide support to their young person, even when struggling to accept or understand their gender identity.
16	Misgendering	TGD young adults	TGD young adults will acquire coping skills and stress management techniques to enhance their mental health and well-being when facing misgendering and other forms of transphobia.
17	Coming Out	TGD young adults	TGD young adults will develop a plan for coming out, prioritizing their safety and personal desire for privacy.
18	Navigating Coming Out	Caregivers	Caregivers will gain consent-based tools to effectively communicate about their young person’s gender with close friends and extended family members, while respecting their young person’s consent.
19	Setting Health Boundaries	TGD young adults	TGD young adults will assess and acquire healthy relationship tools and boundary skills to ensure their safety and emotional well-being while potentially being treated differently.
20	Privacy & Confidentiality	Caregivers	Caregivers will be able to summarize the complexities of confidentiality and consent-based parenting that centers on their TGD young adult.
21	Healthy Peer Relationships	TGD young adults	TGD young adults will learn foundational tools for building healthy and safe relationships, including intimate, platonic, sexual, and romantic relationships. They will also gain the skills to assess their needs and activate a safety plan, including local and national resources for preventing and addressing potential harm or violence.
22	The Talk: Trans Edition	Caregivers	Caregivers will gain skills to effectively discuss sexual health, sexual orientation, and gender identity with their TGD young adult. Caregivers will be able to locate local and national resources to facilitate accurate and safe conversations about dating and sexual health topics with their young person.
23	Managing Self-Harm	TGD young adults	TGD young adults will be able to identify self-harm behaviors and triggers; will develop skills to redirect and seek help from appropriate avenues and resources; and will be able to recognize suicidal ideation and behaviors in themselves and others, when and how to seek help, and learn about appropriate resources to respond to suicidality.
24	Finding Support	Caregivers	Caregivers will build a foundation of skills to foster self-compassion and generate a circle of support for themselves and their young person. They will also gain tools related to self-harm prevention and treatment for their young person.
25	Mental Health Treatment	TGD young adults and caregivers	TGD young adults and their caregivers will recognize ways to find and access mental health care providers who meet their unique needs.
26	Talking with Providers	TGD young adults and caregivers	TGD young adults and their caregivers will learn how to interact with health care providers when seeking regular care.
27	Insurance 101	TGD young adults and caregivers	TGD young adults and their caregivers will learn how to research and navigate insurance systems.
28	Social Transition in Educational Institutions	TGD young adults and caregivers	TGD young adults and caregivers will identify best practices for creating supportive environments at educational institutions and developing gender transition plans.
29	Addressing Bullying & Discrimination	TGD young adults and caregivers	TDG young adults and their caregivers will be prepared to address bullying and harassment in educational institutions and other environments, including cyberbullying, through a law and policy lens.
30	Gender at Work	TGD young adults	TGD young adults will locate resources grounded in law & policy to build their skills in self-advocacy and protection of their rights at work.
31	Addressing Homelessness	Caregivers	Caregivers will identify and name resources available to TGD young adults who face family rejection and will gain the skills to name protective parenting practices to prevent these outcomes.
32	Homelessness	TGD young adults	TGD young adults will identify and name resources available to them when facing family rejection and will gain the skills to create a safety plan with affirming, trustworthy adults in their lives.
33	Looking Ahead	TGD young adults and caregivers	TGD young adults and their caregivers will reflect on their experiences within the app and what they have learned, and set new goals for their continuing journey.

^a^TransHealthGUIDE: Transforming Mental Health for Transgender and Gender-Diverse Young Adults Using Interventions to Drive Equity.

^b^TGD: transgender, nonbinary, and gender diverse.

Additional features of the app include: a goal-setting tool to promote self-efficacy and track progress, a community resources hub offering curated local and national supports, and moderated Q&A forums with TGD community members and expert contributors across various areas of TGD health.

### Study Population and Inclusion and Exclusion Criteria

Two participant groups are being enrolled in the study: (1) TGD young adults ages 18-24 years and (2) adult caregivers of TGD young adults who are enrolled in the study. Inclusion and exclusion criteria for both groups are detailed in Table 2.

**Table 2:** Inclusion and exclusion criteria for TGD^a^ young adults and caregivers in TransHealthGUIDE^b^.

Participant group			Inclusion criteria		Exclusion criteria
**TGD young adults**					
	1	Ages between 18 and 24 years (inclusive)		Ages under 18 years or over 24 years	
	2	A person of TGD experience (transgender, nonbinary, gender diverse, or not cisgender)		Not a person of TGD experience (ie, cisgender)	
	3	Resides in one of the following locations: California, Connecticut, Illinois, Maryland, Massachusetts, New Jersey, New York, Oregon, Vermont, and Washington, DC		Does not reside in the 10 specified locations	
	4	Able to read, write, and communicate in English		Not able to read, write, or communicate in English	
	5	Has access to a smartphone/device or computer with internet connectivity		Does not have access to a smartphone/device or computer with internet connectivity	
	6	Provides informed consent to participate		Does not provide informed consent to participate	
**Caregivers**					
	1	Ages 18 years or older		Ages under 18 years	
	2	A parent or legal guardian of the TGD young adult during their minority		Not a parent or legal guardian of the TGD young adult	
	3	Has a TGD young adult aged 18-24 years enrolled in the study		Does not have a TGD young adult in the study	
	4	Resides in one of the following locations: California, Connecticut, Illinois, Maryland, Massachusetts, New Jersey, New York, Oregon, Vermont, and Washington, DC		Does not reside in the 10 specified locations	
	5	Able to read, write, and communicate in English		Not able to read, write, or communicate in English	
	6	Has access to a smartphone/device or computer with internet connectivity		Does not have access to a smartphone/device or computer with internet connectivity	
	7	Provides informed consent to participate		Does not provide informed consent to participate	

^a^TGD: transgender, nonbinary, and gender diverse.

^b^TransHealthGUIDE: Transforming Mental Health for Transgender and Gender-Diverse Young Adults Using Interventions to Drive Equity.

We aim to recruit 500 participants overall, including 375 TGD young adult participants across 10 US states (listed alphabetically): California, Connecticut, Illinois, Maryland, Massachusetts, New Jersey, New York, Oregon, Vermont, and Washington, DC. Our recruitment goals include at least 50% (250 of 500 participants) identifying as people of color and at least 125 caregivers, resulting in 125 TGD young adult-caregiver pairs.

To maintain the dyadic framework of the intervention, caregivers may only participate if their corresponding TGD young adult is already enrolled. Each TGD young adult is asked to identify a primary caregiver to invite into the study, but young adults can participate in the study without a caregiver.

Caregivers must reside in one of the 10 study states due to legislative and logistical considerations. These locations were selected in consultation with experts that helped identify supportive jurisdictions for TGD young adults, ensuring a foundation of safety and feasibility for the intervention.

For TGD young adults attending college, eligibility is based on their current state of residence during the study period, not their permanent home address. TGD young adults and their caregivers can reside in different states, as long as each lives in one of the 10 eligible states.

### Recruitment

Participants are recruited through a combination of community-based and digital strategies, including caregiver and young adult support groups, TGD-serving organizations, community events, and targeted listservs and newsletters. The study team also leverages personal and professional networks to connect with LGBTQIA+ (lesbian, gay, bisexual, transgender, queer, questioning, intersex, asexual) organizations and individuals.

A key goal of the study is to ensure that at least 50% of enrolled participants are people of color. To support this, we implemented the following targeted recruitment strategies. (1) Community partnerships and outreach: We collaborate with community-based organizations, clinics, and advocacy groups that specifically serve communities of color to raise awareness about the study and encourage participation. (2) Culturally responsive research team: Our team includes trained researchers who have skillsets and experience working with study participants from diverse racial, ethnic, and lived experience backgrounds to help build trust, rapport, and cultural humility in our engagement with communities of from various cultural backgrounds. (3) Diverse research team: Our team includes researchers from diverse racial, ethnic, and lived experience backgrounds to help build trust, rapport, and cultural humility in our engagement with communities of color. (4) Ongoing monitoring and adaptation: We continuously monitor participant demographics and adapt recruitment strategies as needed to meet our diversity and inclusion benchmarks.

### Study Procedures

Potential participants are screened for eligibility online using a study-specific REDCap (Research Electronic Data Capture) screener. Those who appear preliminarily eligible are directed to a contact information form where they provide their name, email, phone number, and preferred method of contact. Study staff then follow up using the contact information provided to schedule an enrollment visit to confirm study eligibility and obtain informed consent. For individuals screened by phone, staff enter the contact information directly into REDCap during the call and schedule the enrollment visit in real time.

All enrollment visits are conducted remotely via a HIPAA (Health Insurance Portability and Accountability Act)-compliant platform, such as Zoom. Participants who meet eligibility criteria are scheduled for a virtual enrollment session during which study staff obtain informed consent using electronic consent procedures. Caregivers are only eligible to enroll if their corresponding TGD young adult has already been enrolled. During the enrollment process, the TGD young adult identifies one primary caregiver to be invited. This ensures that caregiver participation is directly linked by study staff to the corresponding TGD young adult enrollment, preserving the integrity of the dyadic intervention model.

Prior to the start of any study procedures, research staff conduct a comprehensive informed consent process. Participants are informed about the expected length of the session and advised to join from a quiet, private space with a reliable internet or phone connection. Participants are provided a copy of the informed consent form in advance or at the start of the session. A link to the informed consent form in REDCap is sent, and the document is reviewed together during the Zoom call.

Throughout the consent process, staff pause at the end of each major section to summarize key points and check for understanding. Participants are encouraged to ask questions, and staff conduct a verbal comprehension assessment by asking participants to explain back the information they have heard. These checks are repeated after key sections—particularly those on study procedures and risks—and again before signing the form. Any unclear points are rephrased to ensure clarity. Once all questions have been addressed, participants complete the electronic consent form by entering their initials and signature in REDCap. A copy of the signed document is then provided to the participant for their records.

### Intervention Allocation and Randomization

Randomization occurs following completion of the enrollment visit. Participants are randomized in a 1:1 ratio to either the immediate intervention arm or the deferred arm (6-month waitlist control). TGD young adults and their caregivers who enroll as dyads are randomized together as a unit to maintain the dyadic structure of the intervention.

Randomization is conducted using an automated, computer-generated sequence within REDCap. The allocation sequence is concealed, and blinding is maintained at the analyst and investigator levels to reduce bias. Once randomization is complete, study staff notify participants of their group assignment using the participant’s preferred method of contact (eg, email or phone).

Regardless of assignment, all participants receive a curated list of national and state mental health support resources.

### Intervention Delivery

TGD young adults and caregivers assigned to the immediate intervention arm receive full access to the digital app. After informed consent is obtained, participants are emailed a personalized link to download the app and complete registration. Only enrolled participants are able to create an account and access the app content. Participants randomized to the deferred arm also receive immediate access to the app; however, they are initially limited to viewing the Community Resources tab, which includes local, regional, and national resources. All other features are automatically unlocked after 6 months.

The intervention supports dyadic participation, with approximately one-third of TGD young adults expected to participate alongside a caregiver. With mutual consent, TGD young adults and caregivers may choose to link their accounts. All linking is reviewed and performed manually by study staff to ensure participant privacy and data integrity.

For dyads, accounts are manually linked by research staff once both participants have downloaded the app and completed registration. If linking is requested after enrollment, the app includes a feature that allows both participants to independently provide consent for linking. Once consent is confirmed, the study team is notified, and accounts are linked manually. Linking allows for shared activities and progress tracking but is entirely optional. Participants may request to unlink their accounts at any time, and study staff will promptly honor the request. This option, along with the voluntary nature of linking, is clearly communicated during the consent process and outlined in study documentation.

### Measures

#### Self-Reported Surveys

Online self-reported survey assessments are administered at baseline, 6 months, and 12 months (see Figure 1). Both TGD young adults and caregivers complete overlapping and complementary measures. Whenever possible, validated instruments—particularly those previously used with TGD and caregiver populations—were selected to ensure methodological rigor and comparability with existing research. All measures were pilot tested prior to implementation.

A computer-assisted self-interview is used to ensure consistency in data capture (eg, skip patterns and logic checks) and to reduce social desirability bias. Surveys are designed to take approximately 30 minutes to complete.

Survey domains include sociodemographic characteristics (age, race, ethnicity, gender identity, sexual orientation, socioeconomic status, religious affiliation, family composition, urbanicity, and nativity) [36]. Health care access and engagement includes general physical and mental health in the past month [37], health insurance coverage, access to primary care, unmet medical and mental health needs, and use of mental health services (inpatient, outpatient, medications, and support groups). Gender transition and journey includes age of first TGD recognition, types of gender affirmation (social, medical, legal), and family or peer disclosure and support. Family relationships are assessed via validated measures including family communication and satisfaction (Family Adaptability and Cohesion Scale III and IV) [38,39], family acceptance, and family empowerment [40]. Community belonging is measured using the Community Connectedness subscale of the Gender Minority Stress and Resilience Measure [29]. Mental health outcomes include suicidal ideation and attempts (lifetime and past 3 months) using the Columbia Suicide Severity Rating Scale (C-SSRS) [41], nonsuicidal self-injury using the SOARS (Suicidal ideation; Onset, frequency, and methods; Aftercare; Reasons; Stage of change screening and assessment of nonsuicidal self-injury) [42], depressive symptoms using the Patient Health Questionnaire-9 [43], anxiety symptoms using the Generalized Anxiety Disorder-2 [44], gender euphoria (3 items developed from qualitative research) [45], gender dysphoria via the Gender Preoccupation and Stability Questionnaire-2 [46], and substance use risk via the CRAFFT (Car, Relax, Alone, Forget, Friends, Trouble) screener [47]. Psychosocial context includes validated assessments of perceived stress [48], resilience [49], perceived social support [50], social safety [51], and perceived impact of legislative climate [52]. Social context and behavior includes bullying and social media use via questions adapted from the Youth Risk Behavior Surveillance System [53] and civic engagement using the Social Justice Scale [54]. Intervention engagement includes app engagement, acceptability [55,56], and the Net Promoter Score [57]. Caregivers complete additional assessments including the Transgender Knowledge, Attitudes, and Beliefs Scale [58]. TGD young adult-caregiver dyads complete 6 items from the Child-Parent Relationship Scale to assess the perceived impact of joint participation [59].

#### Digital App Usage and Metadata

Digital app usage and metadata information are collected and linked to participants’ survey responses to assess engagement and evaluate the intervention’s impact over time. This linkage enables a nuanced analysis of trends across the study period. The following types of app engagement data are collected: module engagement (number of educational modules completed), interactive features (frequency of engagement with interactive features such as posting, reacting or liking, and responding to posts), time spent (total time spent in the app and within specific modules), dyadic participation (dyad status, frequency of interactions between linked TGD young adult-caregiver pairs), goal-setting feature (usage and frequency of interaction with the goal setting component), log-in frequency (number of log-ins to the app), and technical issues (reports of technical difficulties or user-reported problems.) These metrics support the interpretation of intervention exposure and user experience, and inform potential improvements to future iterations of the app.

### Remuneration

Participants receive a US $25 e-gift card after completing each survey at baseline, 3 months, 6 months, and 12 months, along with a US $25 bonus for completing all surveys, for a total of US $125. Payments are sent via an e-gift card to the participant’s email after each survey is completed.

An e-gift card was chosen as the compensation method due to the remote nature of participant interactions. The US $25 per survey completion amount is based on feedback from the CAB, which deemed this amount appropriate for the study’s context.

### Safety Planning and Response Protocol

We have developed a comprehensive safety plan for participants disclosing significant distress or suicidality, specifically tailored to the needs of our study population. The safety plan outlines clear procedures for monitoring, assessing, and responding to participant distress or suicidality. It was created in consultation with multiple psychiatrists and based on established safety protocols that have been successfully implemented in other studies involving mental health assessments and potential disclosures of suicidality in at-risk populations.

A detailed safety response protocol is provided to participants during the consent process and reiterated at every survey. Participants are required to complete a comprehension quiz during the consenting process to verify their understanding of the safety protocol and response procedures. They are informed that survey submissions will not be monitored in real time and are encouraged to take immediate action if they are experiencing an emergency.

All surveys that intentionally assess suicidality, self-harm, and depression-related survey results are reviewed within a 24-hour window by the research team. Crisis management procedures and clinical assessments, as outlined in the safety protocol, are conducted by a board-certified psychiatrist using appropriate clinical interventions. If a participant’s responses indicate significant distress or suicidality, a board-certified psychiatrist conducts a clinical assessment to determine the appropriate course of action.

Personalized referral information is provided to participants based on their assessment results and clinical recommendations. Participants identified as at risk for harm to themselves or others are referred to appropriate mental health services, including emergency departments or crisis hotlines. The research team does not discourage participants from seeking or continuing mental health care or treatments during the study. Participants are explicitly informed, both during recruitment and throughout the study, that their voluntary participation in the research is not a substitute for other mental health interventions.

### Ethical Considerations

Boston Children’s Hospital (BCH) is the prime grant awardee and serves as the primary site for study operations and implementation. The BCH Institutional Review Board (IRB) serves as the single IRB of record for the study (protocol approval number IRB-P00046532).

The University of Michigan School of Public Health, which houses the methodology and data analysis core, and Massachusetts General Hospital, which serves as the clinical psychiatry and mental health safety core, are designated as relying sites under the single IRB structure. The study is covered by a Certificate of Confidentiality from NIH.

A data safety and monitoring board has been established to oversee the trial’s progress, review safety data, and make recommendations regarding the continuation of the trial based on its evaluation of participant safety and the overall performance of the research.

### Statistical Analysis

#### Power

The study aims to enroll 500 TGD participants between the ages of 18 and 24 years, 125 caregivers, and one-quarter of the young adult sample in a TGD young adult-caregiver dyad. The primary outcome of interest is past 3-month suicidal ideation.

Power calculations determined the sample size needed to detect a significant difference in suicidal ideation between the immediate and deferred intervention arms. To detect an effect size of at least 30% (risk ratio=0.70) in the reduction of suicidal ideation from baseline to 6 months postrandomization, a total sample size of 400 participants (200 per arm) is required to achieve 80% power at a 0.05 significance level.

We selected a 30% effect size threshold based on the assumption that smaller differences would not be clinically meaningful for public health scale-up. To account for an estimated 20% attrition rate, we plan to enroll 500 TGD young adult participants and 125 caregivers at baseline, yielding an anticipated analyzable sample of 400 TGD young adult participants and 100 caregivers.

#### Primary Aim Analyses

The primary outcome is suicidal ideation in the past 3 months, assessed using the 6-item C-SSRS. The C-SSRS demonstrates strong validity, with 100% sensitivity and 99.4% specificity for identifying aborted suicide attempts, and 100% sensitivity and specificity for classifying interrupted and actual suicide attempts, when compared to clinician-administered instruments [41]. The secondary outcome is depressive distress over the past 2 weeks, measured using the Patient Health Questionnaire-9, where a score of ≥10 indicates a positive screen for symptoms of clinically elevated depressive symptoms (88% sensitivity and 88% specificity for detecting major depression) [43].

Before analysis, data will be audited for quality and completeness. Descriptive statistics—including counts, percentages, means, medians, standard deviations, and ranges—will summarize baseline characteristics. Group differences at baseline will be assessed using *t* tests for continuous variables and chi-square tests for categorical variables. Variables that differ significantly between study arms may be included as covariates in subsequent models. All analyses will be conducted using SAS or R statistical software.

To evaluate intervention effectiveness, we will use an intention-to-treat approach. Poisson generalized estimating equations with a log link function will estimate the population-average (marginal) effect of the intervention on suicidal ideation from baseline to 6 months postrandomization [60]. An unstructured correlation structure will be used to account for within-subject correlations across repeated measures (baseline, 3 months, and 6 months).

The primary endpoint analysis will compare the proportion of participants reporting suicidal ideation in the past 3 months between the immediate and deferred (waitlist control) arms. The model will include a common intercept and fixed effects for the intervention arm (immediate vs deferred) and time point (3 vs 6 months). Baseline characteristics that differ significantly between groups and variables related to missingness will be included as covariates, if appropriate.

The primary estimate is the intervention arm assignment. The risk ratio will be estimated by exponentiating the intervention coefficient from the model. Wald tests with robust standard errors will be used to construct 95% CIs and test the primary hypothesis. For TGD young adult-caregiver dyads, we will adjust models to account for dyadic interdependence.

The same analytic approach will be applied to the secondary outcome of past 2-week depressive distress. Statistical significance will be determined at the 0.05 level.

#### Supplemental Analyses

Supplemental analyses will build on the primary analytic models to further explore the intervention’s impact. (1) Dose-response effects: To assess potential dose-response relationships, digital app metadata will be incorporated into the analytic models. Specifically, fixed effects for the number of intervention sessions completed will be added to examine whether greater engagement is associated with greater reductions in suicidal ideation and depressive distress. (2) Heterogeneity of treatment effects: We will examine whether intervention effects vary across subgroups defined by race and ethnicity, gender identity, and geographic region. This will be done by including interaction terms between intervention arm assignment and each subgroup variable in the primary model [61]. Significant interactions will be graphed and interpreted to identify whether certain groups derive greater benefit, which can inform future targeted dissemination and scale-up efforts. (3) Sustained intervention effects: To evaluate whether intervention effects are maintained over time, we will analyze data from participants in the immediate arm who completed both the month 6 and the month 12 assessments. The model will estimate the marginal effect of time on past 3-month suicidal ideation between months 6 and 12. The analytic model will include a common intercept, a fixed effect for time (month 6 vs month 12), and relevant covariates. The risk ratio will be calculated as the exponential of the time coefficient.

#### Secondary Aims Analysis

We will explore whether hypothesized mediators at month 3 and month 6 mediate the relationship between intervention arm assignment and suicidal ideation and depressive distress at month 12. We hypothesize that factors targeted by the intervention—namely, family communication, family satisfaction, family acceptance, and community belonging—will mediate the effects of the intervention on the primary outcomes: past 3-month suicidal ideation and past 2-week depressive distress.

Causal mediation analysis methods [62,63] will be used to estimate the total, direct, and indirect effects of each mediator individually, followed by their combined joint effect [64]. We will also calculate the proportion mediated to determine the extent to which each mediator (or the set of mediators) accounts for the total effect of the intervention. These mediation analyses will be conducted using a series of random effects logistic regression models.

Additionally, we will explore differences in family characteristics based on whether a supportive caregiver could be identified and agreed to participate. These comparisons will offer valuable insights for refining future intervention strategies. TGD young adult-caregiver dyads also complete an additional measure to assess the impact of joint participation on the dyadic relationship. We will further examine potential differences in outcomes between linked and unlinked dyads.

#### Attrition Analysis

Attrition will be monitored monthly using data visualization techniques to track trends over time. Key metrics will include total enrollment, representation of target subgroups (by race, ethnicity, and geographic region), and rates of loss to follow-up. Statistical analyses will be conducted to compare participants retained in the study versus those lost to follow-up, focusing on differences in baseline characteristics.

Patterns of missingness will be systematically examined, and baseline responses will be compared between participants with complete versus incomplete data. To address potential biases due to missing data, modern missing data techniques will be used, with an emphasis on multiple imputation methods [65]. Sensitivity analyses will be conducted to compare results from complete-case analyses with those using multiple imputation, allowing for an assessment of the potential bias introduced by missing data.

## Results

### Community Engagement and CAB Feedback

We have maintained consistent engagement with TGD young adult and caregiver communities since the inception of the project. This collaboration has been instrumental in shaping key aspects of the study. Community feedback influenced several design elements, including the adoption of a 6-month waitlist control condition, which was deemed highly acceptable by our CAB and community partners.

Feedback from the CAB also guided the selection of survey measures—for example, prioritizing self-reported measures of suicidality over clinically administered assessments and incorporating items related to social justice and activism as a recognized form of support. The development of intervention content was similarly shaped by community input, resulting in a diverse mix of text-based information, infographics, interviews with TGD people and caregivers, and simulated scenarios. App features were refined in response to privacy and safety concerns, including the addition of a quick exit button.

We will continue to engage with the CAB throughout the intervention period to gather input and integrate community-informed improvements into the study’s ongoing implementation.

### Outreach

To prepare for recruitment, the outreach and recruitment team developed a comprehensive outreach protocol in collaboration with a multidisciplinary team that included communication and legal experts. Using online searches, referrals, and existing personal and professional networks, the team compiled a master list of over 225 national, state, and local organizations and listservs that serve TGD individuals, the broader LGBTQ+ community, and caregivers (eg, PFLAG).

Beginning on September 12, 2024, the team initiated outreach by contacting 209 organizations (Table 3) and conducting virtual meetings to introduce the project, establish rapport, and address questions. These meetings included the distribution of a recruitment press kit containing digital flyers, listserv-ready email content, and a guide sheet with frequently asked questions.

All organizations that participated in meetings expressed strong enthusiasm for the app, highlighting the urgent need for a resource like this among TGD young adults.

**Table 3:** Outreach activities: location of organizations and listservs engaged by the TransHealthGUIDE^a^ team (N=209).

Location	Outreach activities, n (%)
National^b^	23 (11.0)
California	51 (24.4)
Connecticut	13 (6.2)
Illinois	29 (13.9)
Massachusetts	29 (13.9)
Maryland	21 (10.1)
New Jersey	15 (7.2)
Oregon	14 (6.7)
Vermont	6 (2.9)
Washington, DC	8 (3.8)

^a^TransHealthGUIDE: Transforming Mental Health for Transgender and Gender-Diverse Young Adults Using Interventions to Drive Equity.

^b^National refers to outreach activities in the United States that were not state-specific.

### Recruitment, Screening, and Enrollment

Recruitment and study screening were launched on January 21, 2025. As of May 2, 2025, a total of 149 TGD individuals completed the study screener, with 132 (88.6%) of 149 found eligible (Table 4). This success reflects the strategic and thoughtful recruitment plan led by the outreach team, which included tailored engagement strategies, individualized meetings with community organizations to build trust, and consistent follow-up to support interest and participation.

**Table 4:** Screening, eligibility, and enrollment from January 21 to May 2, 2025.

Characteristic		Participants
**Screening and enrollment cascade,** **n/N (%)**		
	Screened	149/149 (100.0)
	Screen eligible	132/149 (88.6)
	Contacted	101/132 (76.5)
	Eligible and consented	5/101 (4.9)
	Not eligible	17/101 (16.8)
	Eligible, not yet consented	20/101 (19.8)
	Declined before consent	6/101 (5.9)
**Screening by race and ethnicity (n=149)^a^, n (%)**		
	American Indian or Alaskan Native	7 (4.7)
	Asian	15 (10.1)
	Black or African American	13 (8.7)
	Hispanic or Latine	29 (19.5)
	Middle Eastern or North African	4 (2.7)
	Native Hawaiian or Pacific Islander	2 (1.3)
	White	106 (71.1)
	Other	3 (2.0)
	Declined to answer	7 (4.7)
**Eligibility by race and ethnicity (n=132)** ^b^ **, n (%)**		
	American Indian or Alaskan Native	6 (4.5)
	Asian	15 (11.4)
	Black or African American	12 (9.1)
	Hispanic or Latine	27 (20.4)
	Middle Eastern or North African	4 (3.0)
	Native Hawaiian or Pacific Islander	1 (0.8)
	White	103 (78.0)
	Other	3 (2.3)
	Declined to answer	7 (5.3)
**Screening by recruitment source (n=149)^c^, n (%)**		
	Social media	5 (3.3)
	Flyer	9 (6.0)
	Ad	1 (0.7)
	Listserv	62 (41.6)
	Community organization	8 (5.4)
	Health care provider	35 (23.5)
	Health care clinic, hospital, or organization	31 (20.8)
	Friend	8 (5.4)
	Other	8 (5.4)
**Eligibility by recruitment source (n=132)^d^, n (%)**		
	Social media	5 (3.8)
	Flyer	7 (5.3)
	Ad	1 (0.7)
	Listserv	62 (47.0)
	Community organization	7 (5.3)
	Health care provider	33 (25.0)
	Health care clinic, hospital, or organization	31 (23.5)
	Friend	7 (5.3)
	Other	7 (5.3)

^a^Race and ethnicity categories were a check-all-that-apply format. The 149 TGD young adults who screened reported 186 racial and ethnic identities.

^b^Race and ethnicity categories were a check-all-that-apply format. The 132 TGD young adults who were eligible reported 178 racial and ethnic identities.

^c^Recruitment source was a check-all-that-apply format. The 149 TGD young adults who screened reported 167 recruitment sources.

^d^Recruitment source was a check-all-that-apply format. The 132 TGD young adults who were eligible reported 160 recruitment sources.

The number of screened and enrolled participants by race and ethnicity and recruitment source is reported in Table 4. Overall, 28.9% (43/149) of those screened were people of color, and 22% (29/132) of those eligible were people of color. The most reported recruitment source was listservs, followed by health care provider, and health care clinic, hospital, or organization. Enrollment visits began on April 23, 2025. As of May 2, 2025, five TGD young adults have been enrolled.

## Discussion

### Development of a Digital Intervention to Address Mental Health Inequities

Suicidality and depressive distress are pervasive among TGD young adults [11-13], underscoring the urgent need for effective and scalable interventions to address these mental health inequities. Interpersonal and structural stigma—manifesting as minority stress at multiple levels—drives the significantly higher rates of suicidality and depressive distress experienced by TGD young adults compared to their cisgender peers [30,31]. Family functioning and support [5,6,17,18] as well as community connectedness [19-21] have been identified as key protective factors, making them critical targets for public health intervention.

Between 2021 and 2024, we worked in close collaboration with TGD young adults and families to develop TransHealthGUIDE, a digital, app-based educational and support intervention. Through extensive community engagement, formative research, and multidisciplinary input—and in partnership with a professional digital health design and development firm—we created a novel app with 33 content modules grounded in the Social Ecological Suicide Prevention Model [28] and Gender Minority Stress and Resilience Framework [29,30]. This intervention takes a relational approach to mental health and applies a strengths-based lens [32] to enhance education and social support for both TGD young adults and their caregivers.

Our ongoing collaboration with a CAB composed of TGD young adults, caregivers, and health care providers, along with ongoing community partnership activities, ensures that the research is conducted by and for TGD individuals and families. This engagement has strengthened the design and implementation of the RCT, supported recruitment and enrollment feasibility, and enhanced the acceptability and relevance of the intervention content. Our initial launch, with 149 screened and 132 eligible TGD young adults in approximately 3 months, reflects the strong interest in and pressing need for this kind of intervention.

### Limitations

First, as a digital app-based intervention, an inclusion criterion for enrollment is access to a smartphone or computer with internet connectivity. Therefore, individuals with limited or no access to technology will not be represented in the study. Second, our study population is restricted to participants from 10 states selected for their supportive legislative context for TGD individuals. While mental health morbidity and mortality among TGD young adults are high in these locations, this approach restricts our reach to individuals residing in other jurisdictions. In these other jurisdictions, which may include states with states lacking legislative supports for TGD young adults, the experiences of TGD young adults may significantly differ and impact mental health risks. If the RCT demonstrates effectiveness, we will explore opportunities to disseminate the digital app more broadly.

Third, caregivers who participate in this study may be more supportive of their TGD young adults than those who do not participate, given the dyadic nature of involvement. However, based on input from the CAB, we expect heterogeneity in the level and types of support and education that participating caregivers will provide. Additionally, this study will provide valuable insights into dyadic participation and the dynamics of TGD young adult-caregiver relationships.

### Conclusions

Tailored digital app-based interventions for TGD young adults are scalable and have the potential for broad public health impact, given the widespread availability of digital technology. This RCT of a novel digital app-based intervention aims to address suicidality and depressive distress in TGD young adults through education and support, involving both TGD young adults and caregivers. This approach fills a critical gap in existing mental health interventions for this population. The study findings and lessons learned from implementation will contribute to advancing mental health equity for TGD young adults and caregivers.

## Appendix

Multimedia Appendix 1 Peer review report from the ZRG1 MOSS-T - Center for Scientific Review Special Emphasis Panel; RFA-RM-21-021: UNITE Transformative Research to Address Health Disparities and Advance Health Equity (U01) (National Institutes of Health, USA).

## Abbreviations

BCH: Boston Children’s Hospital
CAB: community advisory board
CRAFFT: Car, Relax, Alone, Forget, Friends, Trouble substance use screener
C-SSRS: Columbia Suicide Severity Rating Scale
HIPAA: Health Insurance Portability and Accountability Act
IRB: Institutional Review Board
LGBTQIA+: lesbian, gay, bisexual, transgender, queer, questioning, intersex, asexual
NIH: National Institutes of Health
RCT: randomized controlled trial
REDCap: Research Electronic Data Capture
SOARS: Suicidal ideation; Onset, frequency, and methods; Aftercare; Reasons; Stage of change screening and assessment of nonsuicidal self-injury
TGD: transgender, nonbinary, and gender diverse
TransHealthGUIDE: Transforming Mental Health for Transgender and Gender-Diverse Young Adults Using Interventions to Drive Equity
WPATH: World Professional Association for Transgender Health

## Data Availability

The dataset generated is not publicly available. Deidentified data and a data dictionary will be made available upon reasonable request after the approval of a proposal and the signing of a data use agreement by involved institutions. There is a formal process for external users to request access to data, which involves a review and approval by principal investigators, coinvestigators, and a community advisory board. Further details and forms can be obtained by emailing SLR and RX.
