# Supplementary material for: Transforming Mental Health for Transgender and Gender-Diverse Young Adults Using Interventions to Drive Equity (TransHealthGUIDE): Protocol for a Digital Randomized Controlled Trial
Source: JMIR Res Protoc. 2025 Nov 11;14:e78619. doi: 10.2196/78619 (PMC12648128; doi:10.2196/78619)
Supplement: Multimedia Appendix 1 [file resprot_v14i1e78619_app1.pdf]

SUMMARY STATEMENT

(Privileged Communication)

PROGRAM CONTACT:
Dr Cheryl Boyce
301-435-1070
cboyce@nih.gov

Release Date: **08/16/2021**
Revised Date:

Application Number: **1 U01 DE031552-01**

Principal Investigators (Listed Alphabetically):

ESTRADA, CARLOS R (Contact)
REISNER, SARI

Applicant Organization: BOSTON CHILDREN'S HOSPITAL

Review Group: **ZRG1 MOSS-T (50)**
Center for Scientific Review Special Emphasis Panel
RFA-RM-21-021: UNITE Transformative Research to Address Health Disparities and Advance Health Equity (U01)

Meeting Date: **07/27/2021** ***RFA/PA:*** **RM21-021**
Council: **AUG 2021**
Requested Start: **09/01/2021**
Dual IC(s): **RM, OD**

Project Title: **TransHealthGUIDE: Transforming Health for Gender-Diverse Youth Using Interventions to Drive Equity**
SRG Action: **Impact Score:21**
Next Steps: **Visit https://grants.nih.gov/grants/next_steps.htm**
Human Subjects: **30-Human subjects involved - Certified, no SRG concerns**
Animal Subjects: **10-No live vertebrate animals involved for competing appl.**
Gender: **1A-Both genders, scientifically acceptable**
Minority: **1A-Minorities and non-minorities, scientifically acceptable**
Age: **6A-Children and Adults, scientifically acceptable**

Project Year	Direct Costs Requested	Estimated Total Cost
1	1,030,733	1,832,176
2	1,005,493	1,787,310
3	842,460	1,497,512
4	879,228	1,562,868
5	940,654	1,672,056
TOTAL	4,698,568	8,351,922

1U01DE031552-01 Estrada, Carlos

RESUME AND SUMMARY OF DISCUSSION: The goal of this U01 application is to conduct a hybrid effectiveness-implementation trial to advance equity and reduce health disparities for Transgender and gender diverse (TGD) youth through increased access to gender-affirming care and individual, interpersonal, and community-based changes that increase knowledge and caregiver support to reduce suicidality and improve mental health. Reviewers noted that TGD youth of color face substantial health disparities and oppression that leads to very high rates of mental health disparities, thus the proposed multiphase intervention plan to address this issue is significant and would have a transformative impact in addressing disparities in this population. The investigative team has strong expertise in working with TGD youth, provider competency, medical interventions, mental, behavioral, and sexual health, and family issues, and are well suited to successfully complete the proposed studies. The 4-conceptual framework intervention strategy is robust and comprehensive that includes The Social-Ecological Suicide Prevention Model, Gender Affirmation Model, Healthcare Accessibility Model, and Gender Minority Stress Model. The sample sizes for providers (N = 100) and youth (N = 500) are robust to provide sufficient statistical power for outcome analysis. The study design also provides multiple safeguards for participants and contingency plans for recruitment of participants and uptake of gender-affirming care among providers trained in the intervention. Representatives of various community and health care or health education organizations are also involved in the study. However, few minor weaknesses were identified. Reviewers noted that the intervention will be able to reach only to those TGB youth whose parents/caregivers are open to their transgender status and will not be available to those whose parents are not supportive. It was not clear if two parents need to be involved. The focus on the age range of 13-24 TGD and the rationale for excluding those in late childhood and early adolescence is not clear, since gender transition decisions are taken prior to puberty. There are insufficient details in recruiting/sampling parents/caregivers. However, reviewers noted that the investigative team has recruited a large number of TGD youth in prior studies, so there was confidence that they would be able to recruit the target youth group. There were also some concerns regarding different policies regarding transgender youths in different states and how the investigative team would overcome this barrier. Nonetheless, reviewers noted that these weaknesses are addressable and minor. Thus, the overall enthusiasm of the review panel remained extremely high for this outstanding application.

DESCRIPTION (provided by applicant): Transgender and gender diverse (TGD) youth (ages 13-24), with gender identities differing from their sex assigned at birth, are a growing NIH-designated health disparities population. TGD youth suffer much higher rates of suicide than their cisgender counterparts; over half contemplate suicide in their lifetime. These and other health disparities are compounded for TGD youth who are Black or Hispanic and experience both gender- and race-based stigma and minority stress. Black adolescents are also at an increased risk of suicide; attempts increased 73% from 1991 to 2017. Interventions are needed urgently to improve the mental health of TGD youth, particularly TGD youth who are also racial minorities. We propose a transformative, multi-level intervention to advance equity and reduce health disparities for TGD youth through (1) systemic changes that increase access to gender-affirming care and (2) individual, interpersonal, and community-based changes that increase knowledge and caregiver support, thereby reducing suicidality and improving mental health. Access to gender-affirming care and family support are known to be critical determinants of mental health outcomes for TGD youth, yet no large-scale interventions exist to address these remediable structural and social determinants of health. We focus on five Southeastern states with large Black transgender populations and limited access to gender-affirming care. Our intervention has two components. The first is a provider-training and support program to expand the local workforce of gender-affirming care providers. This entails a free online training course for

continuing education credit and a provider network and pathway for remote synchronous and asynchronous consultations with specialists at centers of excellence. The second is an interactive educational digital platform for TGD youth and caregivers. This provides expert- generated knowledge via educational simulations that are tailored to users' needs identified at the time of intake. Interactive features will promote communication between youth and caregivers and improve access to providers and community resources. We will use a Multiphase Optimization Strategy (MOST) framework to optimize the platform for testing in a hybrid effectiveness-implementation trial with an Immediate Arm (upfront access) and Deferred Arm (access at 6 months). The intervention period for each Arm will last 6 months, followed by an observation period of up to 12 months with continued access. Using validated subscales, we will assess changes in the proportion of individuals reporting suicidal ideation in the prior three months (primary outcome) and in psychological distress and anxiety, caregiver support, and health care empowerment (secondary outcomes), as well as dose effects, heterogeneity of treatment effects across groups, and within- group resilience factors. This innovative, multi-level intervention fulfills a significant unmet need for near-term and sustainable solutions to the health disparities faced by TGD youth and addresses intersecting forms of stigma and inequity to transform mental health outcomes for a highly vulnerable, at-risk youth population.

PUBLIC HEALTH RELEVANCE: Transgender and gender diverse (TGD) youth have a much higher risk of suicide than cisgender youth and suffer health disparities that are frequently compounded by race-based stigma and minority stress. This transformative multi-level intervention targets five Southeastern states with large Black TGD populations but limited access to gender-affirming care to improve the mental health of TGD youth through rapid and sustainable changes in access to care, knowledge, and interpersonal support.

CRITIQUE 1

Significance: 2
Investigator(s): 2
Innovation: 2
Approach: 2
Environment: 1

Overall Impact: The proposed project seeks to address significant health issues for transgender and gender diverse (TGD) youth: suicidality and internalizing psychological distress. TGD youth (and TGD youth of color in particular) face substantial health disparities in suicidality, depression, and anxiety. Although TGD youth are a very small population group, they face substantial oppression in the form of cis-genderism (and potentially other intersecting forms of oppression), which contributes to very high rates of mental health disparities. Further, transgender healthcare is still a relatively new yet growing area of healthcare. The proposed intervention is very innovative with substantial potential for impact to improve multiple outcomes among primary and secondary target groups (i.e., TGD youth, parents/caregivers, and healthcare professionals). The intervention has two components (IC1 and IC2): 1) education/training, consultation, and peer support for healthcare providers regarding transgender health and healthcare; and (2) an interactive digital platform for TGD youth and their parents/caregivers to advance knowledge, skills, communication, support, and empowerment regarding gender transition/affirmation issues in the individual, family, and healthcare contexts. There are several strengths to the approach, including a multiphase intervention development and evaluation plan involving qualitative and quantitative data, rigorous process evaluation and outcome evaluation plans including a RCT to test IC2, up to 7 waves of data collection (depending on target group and outcome), relative large sample sizes (N = 500 for TGD youth), and collaboration with community and healthcare organizations. My enthusiasm for the project was diminished by several concerns related to sampling

and location. TGD youth whose parents/caregivers are not open or accepting about their child's gender identity are unlikely to participate in the study and are the highest need group and more burdened by mental health problems. Further, details were lacking about recruiting/sampling parents/caregivers. The age range of 13-24 for TGD youth participants excludes those in late childhood and early adolescence, which is a critical period for TGD youth were major gender transition/affirmation decisions need to be made (e.g., puberty blockers). Further, youth age 18-24 are in a totally different developmental period than younger youth in terms of biophysical, psychological, educational, and social development, with varying implications for healthcare, social/legal independence, and mental well-being. Finally, the investigators are located in the Boston area, but the study would be conducted in the South. Many of these concerns are addressable. The high need and potential impact of the project remains.

1. Significance:

Strengths

- The proposed project addresses a very important health disparity facing transgender and gender diverse (TGD) youth: suicidality and psychological distress. TGD youth face substantial disparities in suicidality, depression, and anxiety. And, TGD youth of color in particular often report higher rates of suicidal behavior than their White counterparts. This application focuses on TGD youth generally but plans to oversample Black and Latinx/Hispanic youth.
- The intervention to address these problems has two components: (1) education/training, consultation, and peer support for healthcare providers who may serve TGD youth; and (2) an interactive digital platform for TGD youth and their parents/caregivers to advance knowledge, skills, communication, support, and empowerment regarding gender transition/affirmation issues in the individual, family, and healthcare contexts. This intervention has tremendous transformative potential regarding health and social well-being.

Weaknesses

- The intervention may only be able to reach TGD youth with parents/caregivers who are open or accepting about their child's gender identity and any necessary medical, psychological, or social transition/affirmation needs. TGD youth most at risk for suicide are largely those without parents/caregivers who are not open-minded, supportive, and/or accepting.
- The exclusion of TGD youth ages 10-12 limits the potential impact of the intervention. These years right before puberty begins are often critical periods of development for TGD youth in terms of puberty blockers and other potential interventions. Unlike sexual minority youth where sexual orientation issues typically arise during adolescence, for TGD youth, gender identity issues frequently arise during childhood. Certain medical and psychosocial interventions may be needed during childhood or early adolescence to prevent mental distress.
- I understand the focus on metropolitan areas; however, TGD youth in rural areas may be less likely to be reached or participate in the intervention.
- At several points in the application, the researchers note the challenges and disparities facing TGD youth of color in particular; however, the intervention does not seem to specifically address issues related to race or racism that may be intersecting gender issues for TGD youth of color. Therefore, certain intersectional issues may not be specifically addressed that are impactful for TGD communities of color, which could be the focus of targeted intervention sub-components and lead to greater improvements in outcomes for this intersectional population group.

2. Investigator(s):

Strengths

- The investigators have expertise in virtually every area of healthcare for TGD youth: provider competency; medical interventions; mental, behavioral, and sexual health; and family issues. This comprehensive expertise acquired through funded research projects (e.g., NIH and CDC), publications, and practice experience make them ideally positioned to implement the proposed project.
- The research team is multidisciplinary with backgrounds and practice experience in medicine, public health, psychology, and social work. This is a notable strength given that IC1 involves various types of providers.
- Many members of the research team identify as LGBTQ and/or people of color. These positionalities may provide relevant insights to the project and garner support and perceived legitimacy from provider participants and TGD youth and their families.

Weaknesses

- The PI, Dr. Estrada, has biomedical expertise but doesn't appear to have much experience with behavioral science/social medicine research, which is the focus of this project.
- The project team is quite large, though perhaps necessary to develop, implement, and evaluate such a complex, multi-component, novel intervention.
- The investigators have little experience conducting projects in the South, which has important and challenging social, cultural, and political issues that differ from the Northeast where the investigators are based.

3. Innovation:

Strengths

- The multi-level intervention is highly innovative. Intervention component 1, focusing on provider training/education, consultation, and support is needed and would fill a gap for providers who do or could serve TGD youth; having a structured program would be an intentional and likely more effective way to address this vulnerable population, as opposed to current limited, fragmented, and piecemeal approaches.
- Intervention component 2 is even more innovative. A digital, evidence- and expert-informed platform for youth and parents/caregivers on TGD does not exist and is highly needed for both groups. Current resources are scattered across many domains with varying helpfulness and accuracy. A centralized digital platform is a new and needed approach.
- Leveraging technology to provide this intervention from another region is cutting-edge in this digital, tele-communication and tele-learning age.

Weaknesses

- Provider training/education and peer consultation/support is not an innovative intervention approach to improve providers' attitudes, knowledge, and skills; however, the content area (i.e., care for TGD pediatric patients) is what adds novelty to this intervention component.
- IC2 looks primarily psychoeducational with linking to resources. It does not include tele-counseling led by mental health professionals with TDG youth or parents/caregivers individually or dyadically. Youth and family likely need psychological and interpersonal interventions to process the information presented, make informed decisions about care or gender affirmation steps moving forward, addressing disagreements between youth and parents, etc. I assume

that such interventions could and would occur in addition to IC1 and IC2; however, the intervention would be more robust and innovative if it included these additional activities.

4. Approach:

Strengths

- The intervention is informed by 4 conceptual frameworks: The Social-Ecological Suicide Prevention Model, Gender Affirmation Model, Healthcare Accessibility Model, and Gender Minority Stress Model. This conceptual integration provides a robust and comprehensive grounding for the multi-component intervention involving multiple social contexts, health issues, and population groups.
- The project includes an array of quantitative and qualitative data collection and analysis activities. Qualitative data and pilot testing intervention elements will be useful to inform the intervention. The outcome evaluation involves a pretest-posttest cohort evaluation for IC1 and a RCT for IC2 with a waitlist control group. Moderation and mediation analyses may reveal interaction effects for certain groups (e.g., race/ethnicity, gender, state) and illuminate intervention pathways to outcome effects. The many potential analyses to be performed with study data are rigorous and would contribute to new knowledge.
- The investigators will evaluate primary and secondary outcomes as well as hypothesized mediators with measures that have evidence of validity and reliability.
- The sample sizes for providers (N = 100) and youth (N = 500; n = 250 intervention, 250 control) should provide sufficient power for the statistical outcome analyses.
- The intent to treat approach for the RCT outcome evaluation with GEE modeling along with other potential qualitative and quantitative analyses are strengths of this proposal.
- The application includes letters of support from representatives of various community and health care or health education organizations (e.g., MAVEN, Gender Spectrum, Fenway Health, GeMS, Ochsner Gender Clinic, CATCH) that would be important partners in the project and facilitate its success.

Weaknesses

- The conceptual model for the project (Figure 2) is confusing and not a good visualization, lacking distinction in concepts across figure domains and clarity in pathways.
- The researchers propose to use a Multiphase Optimization Strategy (MOST) framework for the study design; however, they fail to articulate 3 distinct phases in their research that would follow the MOST framework: preparation, optimization, and evaluation. Certain research activities seem to fall under both preparation and optimization while other activities fall under optimization and evaluation. In addition, optimization activities should be well-recorded in the process evaluation because they imply modifications to the intervention while it is being delivered, which has implications for fidelity, exposure/dose, and outcome evaluation.
- It was unclear if the provider sample would be stratified by provider type. IC1 includes elements for physicians and other elements for mental/behavioral health professionals.
- The sample size of 500 for TGD youth could be difficult to attain because they are a marginalized, hidden, and hard to reach population. The investigators did not provide any numerical estimates based on whatever limited evidence may be available to support their sampling frame and provide confidence that they can recruit the necessary sample size.

- The sample size for parents/caregivers isn't explicitly stated in the application, though I would assume it is 500 to correspond with the youth sample because IC2 includes dyadic activities. There are outcome measures for parents/caregivers: family communication, satisfaction, and empowerment. The researchers did not address situations where youth who have 2 parents who want to be involved in the intervention and how that would be addressed. Could 2 parents be involved? If just 1, would the youth pick or the parents decide? This is an important issue because typically medical interventions for TGD minors require consent from both parents with legal custody.
- The font size of the tables in the Approach section of the proposal is quite small, making them difficult to read. In the future, please comply with NIH font guidelines for the proposal narrative, tables, and figures.

5. Environment:

Strengths

- Boston Children's Hospital is a world leader in pediatric care and research. Brigham and Women's Hospital is a large research and teaching hospital affiliated with Harvard. Both institutions are leaders in comprehensive transgender health care and research.
- The investigators' institutional environments are resource-rich, and there is no doubt that they will provide all the necessary administrative and scientific supports and resources needed for this project to be successful.

Weaknesses

- The research team is located in the Boston area whereas the study is based in the South. Although the intervention involves online, digital, and tele-communication components, the researchers may be perceived as elite Northeastern outsiders by certain members of the target groups.

Study Timeline:

Strengths

- The proposed timeline is ambitious yet feasible given the size and preparation of the team.

Weaknesses

- The outcome evaluation details for IC1 were not presented in the timeline.

Protections for Human Subjects

Acceptable Risks and/or Adequate Protections

- Acceptable

Data and Safety Monitoring Plan (Applicable for Clinical Trials Only):

Acceptable

- Detailed plans for DSMB were provided

Inclusion Plans

- Sex/Gender: Distribution justified scientifically

- Race/Ethnicity: Distribution justified scientifically
- For NIH-Defined Phase III trials, Plans for valid design and analysis: Not applicable
- Inclusion/Exclusion Based on Age: Distribution not justified scientifically
- I'm not convinced of the exclusion of young people ages 10-12 because of reading ability and internet access. These years right before puberty begins are often critical periods for trans youth in terms of puberty blockers and other potential medical interventions. It's a best practice to write intervention materials at a level that is easy to read by broad swaths of the population. In addition, these days, many kids have their own smartphone, computer, or can access a family computer.

Vertebrate Animals
Not Applicable (No Vertebrate Animals)

Biohazards
Not Applicable (No Biohazards)

Resource Sharing Plans
Acceptable

- Acceptable

Authentication of Key Biological and/or Chemical Resources
Not Applicable (No Relevant Resources)

Budget and Period of Support
Recommend as Requested

CRITIQUE 2

Significance: 1
Investigator(s): 2
Innovation: 2
Approach: 2
Environment: 1

Overall Impact: The proposed hybrid effectiveness-implementation intervention among healthcare providers and transgender and gender diverse (TGD) youth (ages 13-24) and their caregivers is exceptional in terms of its potential to transform the field of gender-affirming care and consequently reduce the risk of suicide and improve mental health among TGD. The novel intervention components will be measured by their impact on tangible outcomes; for 100 clinicians, main outcomes include pre-post assessment of provider knowledge, volume of gender-affirming care encounters, and state-level volume of testosterone prescriptions; for 500 TGD and their caregivers, outcomes include measuring the proportion of youth reporting suicidal ideation over time and psychological distress. Both

intervention components, to be implemented and monitored over 18 months, target 5 U.S. states that are at high risk of both gender-based and race-based inequities. The study framework, design, and analyses are rigorous and well thought-out. The investigative team has highly skilled, diverse members. The proposal includes multiple safeguards for participants and contingency plans for recruitment of participants and uptake of gender-affirming care among providers trained in the intervention. A minor weakness is that the PIs have not collaborated prior to this application. However, it appears that the MPI has collaborated with investigators on the team. If successful, the proposed study could have a high impact by greatly expanding clinical training in gender-affirming care and increasing knowledge and communication between TGD and their caregivers; collectively, these efforts would have a high transformative impact on the mental health and well-being of TGD youth.

1. Significance:

Strengths

- Major: Transgender and gender diverse (TGD) youth (ages 13-24) are more likely to experience social and structural inequity and health disparities that increase their risk of suicide by up to four times greater than cisgender youth.
- Major: In response to rigor of prior research, no large-scale structural interventions have addressed the lack of access to gender-affirming care and family support, which has been shown to be related to poor mental health outcomes among TGD youth.
- Major: Regarding rigor of prior research, systemic interventions to improve family support for TGD youth do not yet exist.
- Major: The proposed hybrid effectiveness-implementation design is highly rigorous, in that it will test the effectiveness, and evaluate the implementation, of a multilevel intervention for provider training in gender-affirming care and development and testing of the digital platform for TGD youth and their families to increase knowledge and caregiver support. If successful, engaging this population could have a high transformative impact in reducing health inequities and suicide risk.
- Major: The conceptual framework of the proposed intervention demonstrates in-depth knowledge of the field and thoughtfulness of the design, which comprises 8 multilevel factors impacting TGD youth and their caregivers and 4 conceptual models that address the provider training and digital platform intervention components.
- Major: Both intervention components target five U.S. states that have limited access, or legislation restricting access, to gender-affirming care.

Weaknesses

- No concerns noted.

2. Investigator(s):

Strengths

- Major: The PIs have collective expertise in management, clinical care, digital health, transgender population health, mental health, substance use, and suicide risk among transgender youth and young adults.
- Major: The study team comprises experts in pediatric medicine (i.e., urology, endocrinology, gynecology) and other clinical aspects of medicine (i.e., plastic and reconstructive surgery, transgender reproductive health), as well as research in gender surgery, sexual orientation and

gender identity, design and delivery of simulation-based immersive videos, health policy and economics, and community-based representation, including a transgender community member and transgender social media influencer.

- Moderate: The study team demonstrates their commitment to diversity by including community members and scholars who represent gender, sexual, and racial/ethnic minorities.
- Moderate: The MPI plan is solid and delineates the roles and responsibilities of the PIs, as well as conflict resolution.

Weaknesses

- Minor: There is no evidence that the PIs have collaborated prior to this application; however, Dr. Reisner has collaborated with two team investigators, representing Boston Children's Hospital.
- Minor: It would have helped to have a more detailed description of the roles and responsibilities of the team (aside from the budget justification) since it is large.

3. Innovation:

Strengths

- Major: The proposed multilevel intervention has the potential to nationally transform training of (for providers) and access to (for TGD youth and their caregivers) gender-affirming care among TGD, which includes Black, Hispanic, and other racial/ethnic minorities.
- Moderate: Developing "peer consultation pathways" that connect local providers with specialists in TGD care is innovative and will add to the potential success of the study.
- Moderate: Simulation-based educational video modules will be developed by clinicians in collaboration with advisory boards of Black, Hispanic, and other TGD individuals and personalized to users' needs is somewhat novel.
- Major: Interactive digital platform features that facilitate communication between TGD youth and their caregivers is highly innovative.

Weaknesses

- No concerns noted.

4. Approach:

Strengths

- Major: For the provider training intervention component, up to 100 providers will be trained in specific areas of gender-affirming care (3 months) and then observed over 18 months to determine whether the training leads to implementation of the study outcomes.
- Major: The primary and secondary outcomes for the provider training intervention component include testing provider knowledge, assessing volume of gender-affirming care encounters (across the five states), and state-level volume of testosterone prescriptions; these measures are tangible and rigorous and can measure impacts from the study over time.
- Major: The proposed rigorous educational digital platform will include educational modules such as simulation-based, knowledge-based, and skills-based videos developed by the study team. Interactions with providers from various health specialties will lend to learning on gender-affirmative care.

- Moderate: Though TGD participants must be fluent in English, the simulation-based video content and static platform content will also be available in Spanish.
- Major: Relevant biological variables (e.g., gender identity; race and ethnicity; age) will be tested for heterogeneity of intervention effects.
- Moderate: Participants will be randomly assigned to the Immediate study arm (educational digital platform) or the Deferred study arm (access to the educational digital platform at 6 months).
- Major: The participant intervention period will be 6 months, followed by study observation of 12 months, which is longer than most research studies and a strength of the design.
- Major: Using a MOST framework to develop, optimize, and test the intervention components demonstrates high rigor.
- Major: Sample size and power estimates and statistical analyses pertaining to the intervention components are robust.
- Moderate: The mixed-methods evaluation, and the qualitative and quantitative process evaluations, of effectiveness and implementation are a strength of the analytic approach.
- Major: Using CME, and covering CME training costs, as an approach to deliver novel gender-affirmative care (across topics including mental health and suicide risk to hormone blockers and surgery assessment to billing codes for procedures) is a great strength of the approach and an innovative way to incentivize providers to receive specialized training.
- Major: There is a comprehensive section on potential challenges and alternate approaches, which includes addressing provider adoption of the training and (regarding the digital platform) making the platform content accessible via computer, tablet, or smartphone.
- Major: regarding clinical trial implementation and procedures, the team mentions safety and ethical issues pertaining to children, provides detailed online consent processes, mentions plans for recruitment accrual monitoring, provides detailed inclusion/exclusion criteria, and accounts for potential differential intervention effects by race and ethnicity, age, gender identity, and state.

Weaknesses

- No major concerns.

5. Environment:

Strengths

- The resources available from Boston Children’s Hospital, Brigham and Women’s Hospital, Mass General, and Harvard are exceptional and include a simulation lab that will be utilized for the proposed study.
- The inclusion of TGD and expert practitioners in gender-affirming care as partners in this initiative, along with integrated feedback from community members, is a strength.
- The strong letters of support from colleagues and key community advocates is a strength.

Weaknesses

- None noted.

Study Timeline:

Strengths

- The timeline is feasible and detailed.

Weaknesses

- None noted.

Protections for Human Subjects

Acceptable Risks and/or Adequate Protections

- The study poses minimal risk to participants; it will be conducted online, is survey-based, and does not require any in-person interactions.

Data and Safety Monitoring Plan (Applicable for Clinical Trials Only):

Acceptable

- The DSMP is comprehensive and addresses the development of the DSMB, DSMB roles and responsibilities, procedures for data review and reporting, protocol for reporting adverse events (including reporting timeframe) and managing participant privacy.

Inclusion Plans

- Sex/Gender: Distribution justified scientifically
- Race/Ethnicity: Distribution justified scientifically
- For NIH-Defined Phase III trials, Plans for valid design and analysis: N/A
- Inclusion/Exclusion Based on Age: Distribution justified scientifically

Vertebrate Animals

Not Applicable (No Vertebrate Animals)

Biohazards

Not Applicable (No Biohazards)

Resource Sharing Plans

Acceptable

Authentication of Key Biological and/or Chemical Resources

Not Applicable (No Relevant Resources)

Budget and Period of Support

Recommend as Requested

CRITIQUE 3

Significance: 1

Investigator(s): 1
Innovation: 2
Approach: 2
Environment: 1

Overall Impact: Transgender and gender diverse (TGD) youth have a much higher risk of suicide than cisgender youth and suffer health disparities that are frequently compounded by race-based stigma and minority stress. This multi-level intervention targets five Southeastern states with large Black TGD populations but limited access to gender-affirming care to improve the mental health of TGD youth through rapid and sustainable changes in access to care, knowledge, and interpersonal support. The study has several strengths including that it addresses a critical barrier which is lack of access to gender-affirming care and family support among Black and Hispanic TGD youth; it proposes a novel multisectoral intervention to address the beforementioned challenges and the potential to produce transformative changes. The project is highly innovative potentially shifting TGD mental health care delivery. A minor to moderate weakness is that the research procedure does not integrate a community engaged approach; nor it engages the community stakeholders in a more active power-sharing role.

1. Significance:

Strengths

- The project addresses critical barrier which is lack of access to gender-affirming care and family support among Black and Hispanic TGD youth, which are critical determinant of mental health and suicidal prevention.
- The project proposes an innovative multisectoral intervention with treatment components targeting Black and Hispanic TGD youth, caregivers, and healthcare providers.
- The proposed research project, if successful, will have a clear transformative potential by tackling multi-factorial structural and social inequities and potentially transforming mental health outcomes among TGD.

Weaknesses

- None noted by the reviewer.

2. Investigator(s):

Strengths

- The research team, and the MPI leadership, is experienced and well qualified to lead this research.

Weaknesses

- None noted by reviewer.

3. Innovation:

Strengths

- The multilevel intervention proposed is novel
- The proposed study, if successful, has the potential to transform TGD mental healthcare delivery and clinical practice.

Weaknesses

- None noted by reviewer.

4. Approach:

Strengths

- The proposed overall strategy and research approach is exceptionally well-reasoned and appropriate to accomplish the proposed aims.
- The of MOST design is a strength, so is the mixed methods process evaluation guided by the RE-AIM and CIFIR frameworks. Together these frameworks increase rigor and ensure a robust approach.
- The study design also appropriately addresses primary and secondary outcome relevant to the hypothesis being tested.
- Challenges, risks, and alternative approaches are addressed and reasonable.
- Recruitment plan is also feasible.
- The power analysis is adequate and statistical analyses are appropriate as well.

Weaknesses

- Community stakeholders are not engaged in a more active partnership role. For instance, the grant mention that investigators “will perform direct outreach to provider who treat patents ages 13-24 in clinics and small practices”, but it seems these outreach activities are intended to facilitate study’s recruitment of providers for the training component of the intervention. Also, they will partner with the MAVEN Project “to expedite provider recruitment.” The grant mentions that investigators will implement a peer consultation pathway between providers in intervention states and specialists at TGD care of excellence, which (to my opinion) is also a strength; however, it would have been best to include these providers as consultants guiding the training development.
- It is unclear how the sample size will be distributed among research sites.
- Although the intervention targets 2 levels (interpersonal and health system), it would have been optimal to also include policy level impact or at least include preliminary data for future scaling-up of the intervention.

5. Environment:

Strengths

- The institutional support, equipment, and other physical resources available to the investigators are adequate for the project proposed and contribute to the probability of success.

Weaknesses

- None noted by reviewer.

Study Timeline:

Strengths

- The study timeline is feasible and well justified.

Weaknesses

- None noted by reviewer.

Protections for Human Subjects

Acceptable Risks and/or Adequate Protections

Data and Safety Monitoring Plan (Applicable for Clinical Trials Only):

Acceptable

Inclusion Plans

- Sex/Gender: Distribution justified scientifically
- Race/Ethnicity: Distribution justified scientifically
- For NIH-Defined Phase III trials, Plans for valid design and analysis: N/A
- Inclusion/Exclusion Based on Age: Distribution justified scientifically

Vertebrate Animals

Not Applicable (No Vertebrate Animals)

Biohazards

Not Applicable (No Biohazards)

Resource Sharing Plans

Acceptable

Authentication of Key Biological and/or Chemical Resources

Not Applicable (No Relevant Resources)

Budget and Period of Support

Recommend as Requested

THE FOLLOWING SECTIONS WERE PREPARED BY THE SCIENTIFIC REVIEW OFFICER TO SUMMARIZE THE OUTCOME OF DISCUSSIONS OF THE REVIEW COMMITTEE, OR REVIEWERS' WRITTEN CRITIQUES, ON THE FOLLOWING ISSUES:

PROTECTION OF HUMAN SUBJECTS: ACCEPTABLE

INCLUSION OF WOMEN PLAN: ACCEPTABLE

INCLUSION OF MINORITIES PLAN: ACCEPTABLE

INCLUSION ACROSS THE LIFESPAN: ACCEPTABLE

COMMITTEE BUDGET RECOMMENDATIONS: The budget was recommended as requested.

Footnotes for 1 U01 DE031552-01; PI Name: Estrada, Carlos R

NIH has modified its policy regarding the receipt of resubmissions (amended applications).See Guide Notice NOT-OD-18-197 at https://grants.nih.gov/grants/guide/notice-files/NOT-OD-18-197.html. The impact/priority score is calculated after discussion of an application by averaging the overall scores (1-9) given by all voting reviewers on the committee and multiplying by 10. The criterion scores are submitted prior to the meeting by the individual reviewers assigned to an application, and are not discussed specifically at the review meeting or calculated into the overall impact score. Some applications also receive a percentile ranking. For details on the review process, see http://grants.nih.gov/grants/peer_review_process.htm#scoring.

MEETING ROSTER

Center for Scientific Review Special Emphasis Panel
CENTER FOR SCIENTIFIC REVIEW
RFA-RM-21-021: UNITE Transformative Research to Address Health Disparities and Advance Health Equity (U01)
ZRG1 MOSS-T (50)
07/27/2021 - 07/28/2021

Notice of NIH Policy to All Applicants: Meeting rosters are provided for information purposes only. Applicant investigators and institutional officials must not communicate directly with study section members about an application before or after the review. Failure to observe this policy will create a serious breach of integrity in the peer review process, and may lead to actions outlined in NOT-OD-14-073 at https://grants.nih.gov/grants/guide/notice-files/NOT-OD-14-073.html, NOT-OD-15-106 at https://grants.nih.gov/grants/guide/notice-files/NOT-OD-15-106.html, and NOT-OD-18-115 at https://grants.nih.gov/grants/guide/notice-files/NOT-OD-18-115.html, including removal of the application from immediate review.

CHAIRPERSON(S)

MURRY, VELMA MCBRIDE, PHD
LOIS AUTREY BETTS CHAIR AND JOE B WYATT
DISTINGUISHED UNIVERSITY PROFESSOR
DEPARTMENT OF HUMAN AND ORGANIZATIONAL DEVELOPMENT
VANDERBILT UNIVERSITY
NASHVILLE, TN 37203

MEMBERS

ABRAIDO-LANZA, ANA F., PHD
PROFESSOR
VICE DEAN SOCIAL AND BEHAVIORAL SCIENCES
SCHOOL OF GLOBAL PUBLIC HEALTH
NEW YORK UNIVERSITY
NEW YORK, NY 10012

ADUNYAH, SAMUEL E, PHD
PROFESSOR AND CHAIRMAN
DEPARTMENT OF BIOCHEMISTRY, CANCER BIOLOGY
NEUROSCIENCE AND PHARMACOLOGY
SCHOOL OF MEDICINE
MEHARRY MEDICAL COLLEGE
NASHVILLE, TN 37208

ARORA, KAVITA SHAH, MD
ASSOCIATE PROFESSOR
METROHEALTH MEDICAL CENTER
CASE WESTERN RESERVE UNIVERSITY
CLEVELAND, OH 44109

BENTLEY-EDWARDS, KEISHA L., PHD
ASSOCIATE DIRECTOR OF RESEARCH, SAMUEL DUBOIS
COOK CENTER ON SOCIAL EQUITY
DUKE UNIVERSITY
DURHAM, NC 27708

BORREGO, MATTHEW, PHD
PROFESSOR
DEPARTMENT OF PHARMACY PRACTICE
AND ADMINISTRATIVE SCIENCES
COLLEGE OF PHARMACY
UNIVERSITY OF NEW MEXICO
ALBUQUERQUE, NM 87131

BRUCE, MARINO A, PHD
CLINICAL PROFESSOR OF BEHAVIORAL AND SOCIAL SCIENCES
COLLEGE OF MEDICINE
UNIVERSITY OF HOUSTON
HOUSTON, TX 77004

BURKE, NANCY J., PHD
DEPARTMENT CHAIR AND PROFESSOR
DEPARTMENT OF PUBLIC HEALTH
SCHOOL OF SOCIAL SCIENCES AND HUMANITIES
UNIVERSITY OF CALIFORNIA, MERCED
MERCED, CA 95343

CASSIDY-BUSHROW, ANDREA E, PHD
ASSOCIATE SCIENTIST AND RESEARCH EPIDEMIOLOGIST
DEPARTMENT OF PUBLIC HEALTH SCIENCES
HENRY FORD HEALTH SYSTEM
DETROIT, MI 48202

CASTRO, EIDA MARIA, PSYD
ASSOCIATE PROFESSOR
DEPARTMENT OF PSYCHIATRY
MENTAL HEALTH DIVISION
SCHOOL OF BEHAVIORAL AND BRAIN SCIENCES
PONCE SCHOOL OF MEDICINE
PONCE, PR 00716

CHAKKALAKAL, ROSETTE J, MD
ASSOCIATE PROFESSOR
DIVISION OF GENERAL INTERNAL MEDICINE AND
PUBLIC HEALTH
MEDICAL CENTER
VANDERBILT UNIVERSITY
NASHVILLE, TN 37235

CHATTERJI, PINKA, PHD
PROFESSOR
ECONOMICS DEPARTMENT
UNIVERSITY AT ALBANY
ALBANY, NY 12222

CHAVEZ, LIGIA M., PHD
ASSOCIATE PROFESSOR
BEHAVIORAL SCIENCES RESEARCH INSTITUTE
UNIVERSITY OF PUERTO RICO
RIO PIEDRAS, PR 00935

COHN, ELIZABETH GROSS, PHD
RUDIN CHAIR AND PROFESSOR OF COMMUNITY-ENGAGED
RESEARCH
ASSOCIATE PROVOST FOR RESEARCH
HUNTER COLLEGE
CITY UNIVERSITY OF NEW YORK
NEW YORK, NY 10065

CUBBIN, CATHERINE, PHD
PROFESSOR AND ASSOCIATE DEAN FOR RESEARCH
STEVE HICKS SCHOOL OF SOCIAL WORK
UNIVERSITY OF TEXAS AT AUSTIN
AUSTIN, TX 78712

DALE, SANNISHA K., PHD
ASSOCIATE PROFESSOR
HEALTH DIVISION
DEPARTMENT OF PSYCHOLOGY
UNIVERSITY OF MIAMI CORAL GABLES
CORAL GABLES, FL 33146

DIAZ, VANESSA ASTRUD, MD
ASSISTANT PROFESSOR
DEPARTMENT OF FAMILY MEDICINE
MEDICAL UNIVERSITY OF SOUTH CAROLINA
CHARLESTON, SC 29425

EHRENTHAL, DEBORAH BETH, MD, MPH
PROFESSOR
DEPARTMENT OF OBSTETRICS & GYNECOLOGY
AND POPULATION HEALTH SCIENCES
UNIVERSITY OF WISCONSIN
SCHOOL OF MEDICINE AND PUBLIC HEALTH
MADISON, WI 53726

ERINOSHO, TEMITOPE O, PHD
ASSOCIATE PROFESSOR
DEPARTMENT OF APPLIED HEALTH SCIENCES
SCHOOL OF PUBLIC HEALTH
INDIANA UNIVERSITY BLOOMINGTON
BLOOMINGTON, IN 27599

GAMAREL, KRISTINE E, PHD
JOHN G. SEARLE ASSISTANT PROFESSOR
DEPARTMENT OF HEALTH BEHAVIOR
AND HEALTH EDUCATION
SCHOOL OF PUBLIC HEALTH
UNIVERSITY OF MICHIGAN
ANN ARBOR, MI 48109

GONZALEZ, CRISTINA M, MD
PROFESSOR
DEPARTMENT OF MEDICINE
MONTEFIORE MEDICAL CENTER
ALBERT EINSTEIN COLLEGE OF MEDICINE
BRONX, NY 10461

HALL, WILLIAM JAMES, PHD
ASSISTANT PROFESSOR
SCHOOL OF SOCIAL WORK
UNIVERSITY OF NORTH CAROLINA CHAPEL HILL
CHAPEL HILL, NC 27599

HICKEN, MARGARET TAKAKO, PHD
RESEARCH ASSOCIATE PROFESSOR
SURVEY RESEARCH CENTER
INSTITUTE FOR SOCIAL RESEARCH
UNIVERSITY OF MICHIGAN
ANN HARBOR, MI 48104

HIRSHFIELD, SABINA, PHD
PRINCIPAL RESEARCH SCIENTIST
DEPARTMENT OF MEDICINE
STAR PROGRAM
SUNY DOWNSTATE HEALTH SCIENCES UNIVERSITY
BROOKLYN, NY 11203

KATZ, MIRA L, PHD
PROFESSOR
DEPARTMENT OF HEALTH BEHAVIOR
AND HEALTH PROMOTION
COLLEGE OF PUBLIC HEALTH
OHIO STATE UNIVERSITY
COLUMBUS, OH 43210

KIM, DANIEL, MD, DRPH
ASSOCIATE PROFESSOR
DEPARTMENT OF HEALTH SCIENCES
BOUVE COLLEGE OF HEALTH SCIENCES
NORTHEASTERN UNIVERSITY
BOSTON, MA 02115

KUNIN-BATSON, ALICIA S, PHD
ASSISTANT PROFESSOR
DEPARTMENT OF PEDIATRICS
UNIVERSITY OF MINNESOTA MEDICAL SCHOOL
MINNEAPOLIS, MN 55414

LEONE, LUCIA A, PHD
ASSOCIATE PROFESSOR
DEPARTMENT OF COMMUNITY HEALTH AND HEALTH BEHAVIOR
SCHOOL OF PUBLIC HEALTH AND HEALTH PROFESSIONS
STATE UNIVERSITY OF NEW YORK AT BUFFALO
BUFFALO, NY 14214

LI, YUE, PHD
PROFESSOR OF PUBLIC HEALTH SCIENCES
DIRECTOR, HEALTH SERVICES RESEARCH & POLICY (HSRP)
DIVISION OF HEALTH POLICY AND OUTCOMES RESEARCH
DEPARTMENT OF PUBLIC HEALTH SCIENCES
UNIVERSITY OF ROCHESTER MEDICAL CENTER
ROCHESTER, NY 14642

MARTINEZ, MARIA ELENA, PHD
SAM M. WALTON ENDOWED CHAIR FOR CANCER RESEARCH
PROFESSOR AND ASSOCIATE DIRECTOR
POPULATION SCIENCES, DISPARITIES
AND COMMUNITY ENGAGEMENT
UC SAN DIEGO MOORES CANCER CENTER
LA JOLLA, CA 92093

MARTINEZ, PRISCILLA, PHD
ASSOCIATE SCIENTIST
ALCOHOL RESEARCH GROUP
PUBLIC HEALTH INSTITUTE
EMERYVILLE, CA 94608

MCDONOUGH, IAN, PHD
ASSOCIATE PROFESSOR
DEPARTMENT OF PSYCHOLOGY
COLLEGE OF ARTS AND SCIENCES
UNIVERSITY OF ALABAMA, TUSCALOOSA
TUSCALOOSA, AL 35487

MITCHELL, SUZANNE E, MD
ASSOCIATE PROFESSOR
DEPARTMENT OF FAMILY MEDICINE
SCHOOL OF MEDICINE
BOSTON UNIVERSITY
BOSTON, MA 02118

MITSIADES, NICHOLAS, MD, PHD
ASSOCIATE PROFESSOR
MEDICINE-HEMATOLOGY AND ONCOLOGY
DEPARTMENT OF MOLECULAR AND CELLULAR BIOLOGY
COLLEGE OF MEDICINE
BAYLOR COLLEGE OF MEDICINE
HOUSTON, TX 77030

MOSKOWITZ, JUDITH T, PHD
PROFESSOR
DEPARTMENT OF MEDICAL SOCIAL SCIENCES
FEINBERG SCHOOL OF MEDICINE
NORTHWESTERN UNIVERSITY
CHICAGO, IL 60611

ODERO-MARAH, VALERIE, PHD
PROFESSOR & ASSISTANT DIRECTOR OF RESEARCH
DEPARTMENT OF BIOLOGICAL SCIENCES
CENTER FOR CANCER RESEARCH AND THERAPEUTIC DEVELOPMENT
CLARK ATLANTA UNIVERSITY
ATLANTA, GA 30314

PRESS, VALERIE G, MD
ASSOCIATE PROFESSOR
DEPARTMENTS OF MEDICINE AND PEDIATRICS
UNIVERSITY OF CHICAGO
CHICAGO, IL 60637

RANGACHARI, PAVANI, PHD
PROFESSOR
DEPARTMENT OF INTERDISCIPLINARY HEALTH SCIENCES
DEPARTMENT OF FAMILY MEDICINE (MCG)
THE GRADUATE SCHOOL
AUGUSTA UNIVERSITY
AUGUSTA, GA 30912

RICKS-SANTI, LUISEL J., PHD
DIRECTOR
CANCER RESEARCH CENTER
HAMPTON UNIVERSITY
HAMPTON, VA 23668

ROTE, SUNSHINE MARIE, PHD
ASSOCIATE PROFESSOR
KENT SCHOOL OF SOCIAL WORK
UNIVERSITY OF LOUISVILLE
LOUISVILLE, KY 40292

SCHEIM, AYDEN I, PHD
ASSISTANT PROFESSOR
EPIDEMIOLOGY AND BIOSTATISTICS
SCHOOL OF PUBLIC HEALTH
DREXEL UNIVERSITY
PHILADELPHIA, PA 19104

SHARIFF-MARCO, SALMA, PHD
ASSOCIATE PROFESSOR
DEPARTMENT OF EPIDEMIOLOGY AND BIOSTATISTICS
HELEN DILLER FAMILY COMPREHENSIVE CANCER CENTER
GREATER BAY AREA CANCER REGISTRY (GBACR)
UNIVERSITY OF CALIFORNIA, SAN FRANCISCO
SAN FRANCISCO, CA 94158

SONIK, RAJAN ANTHONY, JD, PHD
DIRECTOR OF RESEARCH
ALTAMED HEALTH SERVICES CORPORATION
LOS ANGELES, CA 90040

TEHRANIFAR, PARISA, DPH
ASSOCIATE PROFESSOR
DEPARTMENT OF EPIDEMIOLOGY
MAILMAN SCHOOL OF PUBLIC HEALTH
COLUMBIA UNIVERSITY
NEW YORK, NY 10032

TOBIN, KARIN E, PHD
ASSOCIATE PROFESSOR
DEPARTMENT OF HEALTH, BEHAVIOR, AND SOCIETY
BLOOMBERG SCHOOL OF PUBLIC HEALTH
JOHNS HOPKINS UNIVERSITY
BALTIMORE, MD 21205

TULU, BENGISU, PHD
PROFESSOR
BUSINESS SCHOOL
WORCESTER POLYTECHNIC INSTITUTE
WORCESTER, MA 01609

VUPPUTURI, SUMA, PHD
SENIOR RESEARCH SCIENTIST
MID-ATLANTIC PERMANENTE RESEARCH INSTITUTE
KAISER PERMANENTE MID-ATLANTIC
ROCKVILLE, MD 20852

WANG, JUNLING NONE, PHD
PROFESSOR AND VICE CHAIR FOR RESEARCH
DEPARTMENT OF CLINICAL PHARMACY
AND TRANSLATIONAL SCIENCE
COLLEGE OF PHARMACY
UNIVERSITY OF TENNESSEE HEALTH SCIENCE CENTER
MEMPHIS, TN 38163

WESCOTT, SIOBHAN M, MD
PROFESSOR & DIRECTOR OF AMERICAN INDIAN HEALTH PROGRAM
COLLEGE OF PUBLIC HEALTH
UNIVERSITY OF NEBRASKA MEDICAL CENTER
GRAND FORKS, ND 58202

WHITT-GLOVER, MELICIA C, PHD
PRESIDENT AND CHIEF EXECUTIVE OFFICER
GRAMERCY RESEARCH GROUP, LLC
ADJUNCT ASSOCIATE PROFESSOR
WAKE FOREST SCHOOL OF MEDICINE
WINSTON-SALEM, NC 27106

WILLIAMS, DONNA L., DRPH
PROFESSOR
DEPARTMENT OF BEHAVIORAL HEALTH SCIENCES
LOUISIANA COMPREHENSIVE CANCER CONTROL PROGRAMS
HEALTH SCIENCE CENTER
LOUISIANA STATE UNIVERSITY, NEW ORLEANS
NEW ORLEANS, LA 70112

WILLIAMS, JONI STROM, MD
ASSOCIATE PROFESSOR
DEPARTMENT OF MEDICINE
DIVISION OF GENERAL INTERNAL MEDICINE
MEDICAL COLLEGE OF WISCONSIN
WAUWATOSA, WI 53226

SCIENTIFIC REVIEW OFFICER

BEHERA, ARUNA K, PHD
SCIENTIFIC REVIEW OFFICER
CENTER FOR SCIENTIFIC REVIEW
NATIONAL INSTITUTES OF HEALTH
BETHESDA, MD 20892

EXTRAMURAL SUPPORT ASSISTANT

ROBINSON, LYNDA K., BS
LEAD GRANTS TECHNICAL ASSISTANT
CENTER FOR SCIENTIFIC REVIEW
NATIONAL INSTITUTES OF HEALTH
BETHESDA, MD 20892

OTHER REVIEW STAFF

FLEMING, LIA CAROLINE, MPH
REVIEW ANALYST
CENTER FOR SCIENTIFIC REVIEW
NATIONAL INSTITUES OF HEALTH
BETHESDA, MD 20892

HONG, SEO YOUNG, MPH
REVIEW ANALYST
CENTER FOR SCIENTIFIC REVIEW
NATIONAL INSTITTUES OF HEALTH
BETHESDA, MD 20892

JAIN, ADITI, BS, MPH
REVIEW ANALYST
CENTER FOR SCIENTIFIC REVIEW
NATIONAL INSTITUTES OF HEALTH
BETHESDA, MD 20892

Consultants are required to absent themselves from the room during the review of any application if their presence would constitute or appear to constitute a conflict of interest.
